# The agreement between workers and within workers in regard to occupational exposure to mercury in dental practice assessed from a questionnaire and an interview

**DOI:** 10.1186/1745-6673-6-8

**Published:** 2011-03-23

**Authors:** Kristin Svendsen, Bjørn Hilt

**Affiliations:** 1 Department of Industrial Economics and Technology Management, Norwegian University of Science and Technology. Trondheim, Norway; 2 Department of Occupational Medicine, St Olavs University Hospital in Trondheim, Norway; 3 Department of Public Health and General Practice, Faculty of Medicine, Norwegian University of Science and Technology, Trondheim, Norway

**Keywords:** epidemiology, retrospective exposure assessment, misclassification

## Abstract

**Background:**

The correct assessment and classification of exposure is essential in epidemiology. The validity of exposure data obtained by the use of questionnaires is, however, seldom evaluated. When conducting a study on the possible health effects from mercury exposure in dental practice, we compared answers on exposure from a job-specific questionnaire with answers to the same questions given at an interview 6 to 18 months later.

**Methods:**

We examined the concordance between workers by comparing answers to the questionnaire given by persons working in the same clinics during the same time spans and the agreement within workers by comparing answers to the same questions from a questionnaire and from an interview. Other aims were to see if there was a difference in the answers to the questionnaire across job titles and to study the impact of missing information on the response rate in a detailed questionnaire.

**Results:**

There was a marked difference between the pairs of employees working in the same clinic regarding the start and termination years for the different preparation methods, and this was partly independent of their occupation. Kappa values for using different preparation methods in the questionnaire and at the interview varied between 0.41(moderate) to 0.88(very good).

**Conclusions:**

The results of this study indicate that a mailed questionnaire will cause misclassification of exposure. The observed occurrence of false positive exposure classifications from the questionnaire compared to the interview was higher than for false negative. This is important and may result in serious bias if the prevalence of exposure is low. Due to missing information, detailed questionnaires may also be inefficient if the goal is to construct exposure measures from combinations of several answers in the questionnaire.

## Introduction

Exposure assessment based on questionnaires can introduce different biases involving both differential and nondifferential misclassification. Differential misclassification of the exposure occurs when the likelihood of being classified as exposed is dependent on the outcome, while nondifferential misclassification is a question of sensitivity and specificity of the tool used. In epidemiological studies, differential misclassification may bias an observed relative risk in both directions, while nondifferential misclassification most often biases the relative risk towards the null effect [1,2]. Detailed job-specific questionnaires are considered to differentiate fairly well between high and low exposed workers [3]. It has, however, also been shown that self-reported exposure histories obtained by mailed questionnaires tend to underreport occupational exposure [4,5]. Earlier studies have also shown that while both the sensitivity and specificity of self-reported questionnaires may be satisfactory, missing information, rather than erroneous reporting of exposure, is often the most significant source of misclassification[6]. An extensive review has been published on the literature on the validity and reliability of common case-control exposure assessment methods, including self reported exposure[7].

When working on a retrospective study on the possible late effects from previous exposure to mercury in dental personnel, we developed a model for calculating a relative exposure score based on the answers from a detailed job-specific questionnaire. This score was intended to represent a value for the individual cumulative exposure to metallic mercury from previous work with dental amalgam. In the questionnaire, the respondents were asked in what years they performed the different kinds of amalgam preparations and how many treatments they performed each week. They were also asked how the amalgam was handled, if there were spills, and details about the work premises. The questionnaire as such and how the relative exposure score was calculated have been described in detail elsewhere [8]. Between 6 and 18 months after having responded to the questionnaire, a selection of the study population was interviewed on the same topics.

The aim of this part of the study was to investigate:

1) Do respondents answer the same about previous exposures on two different occasions and with different methods, one by means of a mailed questionnaire and one by means of an interview?

2) Do respondents who had worked in the same clinic at the same time answer the same regarding exposures?

3) Are there differences in agreement between the answers across professional groups (between two dental nurses who have worked in the same clinic and a dental nurse and a dentist)?

4) Does an attempt to make an exposure score from several detailed questions in a questionnaire reduce the number of valid respondents compared to a simple yes-no question on exposure?

## Method

The questionnaire study was conducted during the spring of 2006 in subjects who had worked in the dental health services in three counties in the central part of Norway. From both private and public dental clinics we obtained lists of 2,247 previous and current dental personnel, born between 1913 and 1985, who were candidates for inclusion in the study. All of them received a mailed questionnaire to be returned in a prepaid envelope. The subgroups studied in this investigation were derived from the 1,193 respondents from this primary survey. The questionnaire part of the study is described in a former publication [9].

### Questionnaire for the primary study group

In the mailed questionnaire the participants were asked about their vocational title, what clinics they had worked at, and during what time periods. There were also asked if they had ever worked with the various methods for amalgam preparation that had been common in Norway; i) the use of copper amalgam that was heated in a small pan and used frequently for the restoration of deciduous teeth, ii) weighing and mixing mercury and alloy manually in a mortar, iii) the production of self-made capsules, iv) the use of a semi-automatic mixer (Dentomat), and, v) the use of pre-produced capsules. The questionnaire had been tested on a group of dentists and dental nurses to ensure that the questions were correct and accurate. Copper amalgam consisted of 70% mercury and 30% copper, the amalgam used in the other preparation methods consisted of 50% mercury and an alloy with 70% silver, 25% tin, 1-6% copper, and 0-2% zinc. These preparation methods have been described in detail in another publication [8]. The participants were also asked when they started and when they stopped using the different preparation methods, and how many patients they treated each week with each particular method. If a Dentomat or similar mixing device had been used, the participants were asked how often it was filled, if there had been frequent spills of mercury, whether the amalgam was soft or firm, and whether they were the ones who filled the Dentomat with mercury and alloy. In the questionnaire they were also asked about spills of mercury in general, flooring, ventilation, and other characteristics of the places where the participants had worked. Some of the questions that were assumed to be of most significance for describing the personal exposure to mercury were selected for use in this part of the study. The questions selected for use in this part of the study are given in Table 1.

**Table 1:** Questions used in the questionnaire and for the interview with an occupational physician.

Questions used from the questionnaire	Questions in the interview guide
Have you used copper amalgam? yes- no	Have you ever worked with copper amalgam?
If yes:	In that case, to what extent and for how many years?
In what time period did you do this:	
What year did you start using copper amalgam?	
In what year did all your use of copper amalgam end*	
Have you worked with amalgam that was manually mixed in a mortar? yes-no	Have you ever manually weighted mercury and alloy and mixed it in a mortar?
If yes:	In that case, when and for how many years?
From year...to year...	
Have you mixed amalgam in a Dentomat? Yes-no	Did you use a Dentomat (semi-automatic device) to prepare amalgam?
If yes:	In that case, when and for how many years?
In what time period did you do this:	
From year... to year....*.	
Was the Dentomat adjusted to produce soft or firm amalgam? Soft.... Firm...*.	
How often was the Dentomat filled?*	
More often than once a week.*	
Once a week.*	
Once every second week.*	
More seldom than once every second week.*	

Questions from the questionnaire that was used in the between worker study are marked with *.

### Identification of the subjects who had worked at the same clinics during the same time - the between worker study

To find pairs of participants from the primary study group who had worked at the same clinic at the same time, we focused on responders from smaller communities. The selection was based on the current postal code of the responders' residence. For this work we chose to concentrate on smaller cities and community centers in order to increase the possibility that persons still lived in the city/community where they had worked. By the use of this restriction we also wanted to increase the probability that two or more participants residing in such a small city/community had in fact worked in the same clinic, as smaller communities in Norway tend to have only one dental clinic, while bigger cities usually have several public clinics and smaller, private clinics. As the name of the clinic was written in a free text field in the questionnaire, the individual responses were then examined one by one in order to create a list of clinics with pairs of employees working in the same clinic. Finally, we cropped the list to include only those pairs who had worked at least between 1980 and 1990 because this was a decade when the two most important preparation methods regarding exposure to mercury (using copper amalgam and using a Dentomat) were still in use, and when many dental clinics changed from these methods to more modern ones (e.g. pre-produced capsules). Most of the persons who had worked during the years 1980 to 1990 also had worked in the same clinic several years before and/or after this time period.

This procedure gave us 48 pairs from 23 clinics with 2 persons or more who had worked there at least in the years between 1980 to1990. The answers that were compared within these pairs regarded details on how, when, and how often they used the two preparation methods for amalgam; the copper amalgam and the Dentomat. The question about soft or firm amalgam had only two outcomes, while the question on how often the Dentomat was filled had four possible answers; i) more often than once a week, ii) once a week, iii) once every second week, iv) and more seldom than every second week. The term "partly agree" refers to answers next to each other on the scale i-iv and the term "partly disagree" refers to answers two positions from each other on the scale i-iv. All these questions are related to conditions in the clinic and not to individual work practice. As all pairs had not answered all the relevant questions, the number of comparisons was limited. For this part of the study it was difficult to cover the whole time period for the different preparation methods. As most of the clinics stopped using manually mixing of amalgam in the mortar and started using copper amalgam before 1980, these questions were excluded in the between worker analysis. The questions used in the between worker study are given in Table 1, and marked with an asterisk. We ascertained from the data that the person had started to work in the specific clinic before the year when they reported having started to use a specific method. In the same way; we ascertained that the person was still working in the same clinic after the year they reported having ended using the specific method. Thus, the methods used in the same clinic at the same time, and the year for the start and end of the use of a method should have been the same for the persons who worked there.

### Interview by an occupational health physician - the within worker study

From the 822 female respondents in the original questionnaire study, we intended to invite 100 subjects under the age of 70 for further neuropsychological investigations. Our capacity to do extensive investigations of each person was the main factor that limited the number of participants to be included. In the end we had 95 subjects who showed up for the investigations. Among them, four had to be excluded, three because of other CNS diseases (two with brain tumours and one with sequelae after a cerebral stroke), and one because she was over 70 years of age. As a consequence, we ended up with 91 participants who were both interviewed and underwent neuropsychological investigation.

This interview with an occupational health physician took place between 6 and 18 months after they had responded to the questionnaire. During the interview the occupational physician inquired about the occupational history of the participants and also asked the same questions as in the original questionnaire in regard to the use of the different preparation methods for amalgam i) heated copper amalgam, ii) manually weighting and mixing in a mortar and iii) a Dentomat. The respondents were also asked how long they had used the 3 preparation methods. The questions in the interview guide are given in Table 1.

### Ethical considerations

The study was approved by the ethical committee for medical research in Central Norway, and we had a licence for personal registrations from the Norwegian Social Science Data Services. The conduct of the study was deemed to be in accord with the Helsinki declaration on medical research ethics.

### Statistical analysis

Data were registered and analyzed with the data program Statistical Package for Social Science version 14.0 (SPSS) (SPSS Inc., Chicago, IL, USA). The concordance between participants who had worked in the same clinic regarding the years the participants started and ended the use of copper amalgam and Dentomat was analyzed by calculating the mean difference from the absolute values of the difference between the pairs, standard deviation of the mean difference, and the 10, 50 and 90 percentile of the difference between the pairs of dental personnel in the same clinic, between those pairs that consisted of two dental nurses or two dentists and the pairs that consisted of one dental nurse or one dentist [10]. The within worker agreement on the given number of years using the different treatment methods from the questionnaire and the interview respectively was also calculated in the same way. Cohen's kappa statistic was used as a measure of the within worker agreement between the questionnaire and the interview for the question as to whether they had ever used the three different treatment methods.

## Results

### The between worker study

For the between worker study, 68 persons who had worked in dental health care between 1980 and 1990 were available. They constituted 48 pairs. Among these persons, there were 43 female dental nurses 6 female dentists and 19 male dentists. The mean age at the time of the questionnaire study for the females was 60.6 (SD 8.3) and for the men 63.2 (SD 5.9). The year of first employment in dental care ranged from 1955 to 1980.

Table 2 shows the concordance between the pairs of dental personnel (all), the pairs of two dental nurses, the pairs of one dental nurse and one dentist, and the pairs of two dentists in the same clinic at the same time with regard to the year when all the use of copper amalgam had ended, the year the use of Dentomat started, and the year the use of the Dentomat ended. The concordance is given as the mean difference between the pairs for the year of starting or ending the two preparation methods. The 10, 50 and 90 percentile for the difference between the pairs is also given. The highest mean differences appeared for the year when the use of copper amalgam ended.

**Table 2:** Concordance between workers in regard to information about time.

Question	Comparisons (number of pairs)	mean	Mean Difference years	Std of the mean difference	10 percentile	50 percentile	90 percentile
The year all use of copper amalgam ended	All (19)	1984	7.3	6.5	0.0	5.0	20.0
	Both dental nurses (13)		9.0	7.0	1.0	7.0	21.8
	One dentist and one dental nurse ( 6)		3.5	3.1	0.0	4.0	8.0
The year the use of the Dentomat started	All (24)	1976	4.7	5.8	0.0	2.5	15.5
	Both dental nurses (10)		4.2	4.9	0.0	4.0	14.4
	One dentist and one dental nurse (13)		5.5	6.7	0.0	2.0	18.4
The year the use of the Dentomat ended.	All (26)	1995	5.5	4.9	0.0	5.0	12.9
	Both dental nurses (12)		7.4	4.9	0.6	7.0	15.7
	One dentist and one dental nurse (12)		3.5	4.2	0.0	4.5	9.4
	Both dentists (2)		6.5	4.9			

Concordance expressed as the mean of the absolute difference and its standard deviation between people working in the same clinic in the same time spans. The 10, 50 and 90 percentiles for the differences between pairs are also given.

Table 3 shows the percent of concordance regarding the questions about the use of the Dentomat between the pairs who had worked in the same clinics at the same time. Regarding the question on how often the Dentomat was filled, 70% of the pairs agreed or almost agreed. The mean difference between the number of years working with a Dentomat between workers in the same clinic at the same time was 6.0 (SD 6.0) for the 14 available pairs.

**Table 3:** Percent of concordance between pairs that worked in the same clinic in the same years and during 1980 to 1990.

Question	Comparisons (number of pairs)	Percent of pairs that			
		
		agree	Partly agree^1^	Partly Disagree^2^	Total disagree
Did the Dentomat produce soft or firm amalgam?	All (31)	64.5			35.5
	both dental nurses (16)	68.8			31.2
	one dentist and one dental nurse (14)	57.1			42.9
	both dentists (1)	100			
How often was the Dentomat filled?	All (30)	23.3	46.7	26.7	3.3
	both dental nurses (15)	33.3	46.7	20	0
	one dentist and one dental nurse (15)	13.3	46.7	33.3	6.7

The question on soft or firm amalgam had a dichotomous outcome, while the question on how often the Dentomat was filled had the answers; more often than once a week, once a week, once every second week, and more seldom than every second week.

^1^Answers next to each other on a scale i-iv

^2^Answers two positions from each other on a scale i-iv

### The within worker study

The participants in the within worker study were all female: 16 dentists, 70 dental nurses and 5 from other occupational groups in dental care. Their mean age at the time when the questionnaire study was conducted was 56.9(SD 6.4). The year of first employment in dental care ranged from 1954 to 1996. Table 4 shows the within worker agreement expressed as kappa statistics between the questionnaire and the interview in regard to ever having used copper amalgam, performed manual mixing in a mortar, and used a Dentomat. Taking the answers from the interview on these questions as the true answer and comparing this to the similar answers from the questionnaires, the sensitivity of the questionnaire was 0.92, 0.83 and 0.92 for the three different preparation methods while the specificity was 0.79, 0.55 and 0.42 respectively.

**Table 4:** Kappa statistics for the same answers in the questionnaire and the interview.

Question	N	Yes from interview	Yes from questionnaire	kappa	95% conf intervals
Yes-no have ever used copper amalgam	84	51	52	0.88	0.77-0.99
Yes-no have ever used a mortar	85	28	48	0.41	0.24-0.58
Yes-no have ever used a Dentomat	85	76	77	0.54	0.24-0.84

Results for non-missing respondents

Table 5 shows the number of years the participants reported they had used the different preparation methods, and the mean difference between the questionnaire and the interview for the number of years for those who had used the different preparation methods. In addition the 10, 50 and 90 percentile for the differences are given.

**Table 5:** Agreement within workers for the number of years they had used the three treatment methods, heated copper amalgam, a mortar and a Dentomat.

Question	number of comparisons	From interview	From questionnaire	Mean difference	Std of the mean difference	10 percentile	50 percentile	90 percentile
Number of years with copper amalgam	47	7.6(5.2)	9.1(5.4)	3.7	3.5	0	2.0	9.2
Number of years with use of a mortar	22	6.6(4.2)	8.8(7.2)	5.8	5.9	0	5.0	14.4
Number of years with use of a Dentomat	61	16.6(8.5)	16.9(9.1)	5.9	5.6	0	4.0	15.8

The difference is the mean difference for the absolute value of the differences on the number of years given at the interview and the number of years given in the questionnaire for those who had used that method. The 10, 50 and 90 percentiles for the differences between the interview and the questionnaire are also given.

When we analyze the answers at the interview for the persons with missing information regarding the use of the different preparation methods in the questionnaire, we see that two out of seven who had missing information on the use of copper amalgam from the questionnaire answered at the interview that they had used this method. For the use of a mortar, the answer was positive for two out of six who had missing information in the questionnaire and for the use of a Dentomat the answer was positive for three out of six. This means that between 29 to 50% of the missing answers in the questionnaire may be "yes".

When looking at the answers to the questionnaires in the primary study group on the use of the three amalgam preparation methods it turned out that only 71.8% of those who had answered "yes" to the question which asked if they had used the copper amalgam method had completed the information on the topic in regard to both how many years they had used the method and how many patients they had treated each week with this method. The similar value for using a mortar was 61.8% and for using a Dentomat it was 52.6%. All in all, only 47.9% of the dental personnel from the primary study group had given all the answers in the questionnaire that were needed in order to calculate the relative exposure score. If we had only used the yes or no answers on the questionnaire for the 4 preparation methods (copper amalgam, mixing manually in mortar, make own capsules or use a Dentomat), the response rate from the questionnaire would have been 81.9%.

## Discussion

In our primary study, an effort was invested in an extensive job-specific questionnaire sent to the dental personnel in order to obtain as accurate information on the cumulative exposure to mercury as possible. However, such a detailed questionnaire turned out to be rather inefficient, as less than 50% of the respondents in the primary study group had answered all the questions that were necessary for the calculation of the exposure score. When studying the validity of the answers from the questionnaire compared to the answers in an interview regarded as the "truth", it became apparent that the agreement, the sensitivity, and the specificity varied for the different questions. In particular, the specificity of the questionnaire compared to the interview was somewhat low. But, using the interview as a "gold standard" may not be correct. We supposed however, that the answers given during the interview with the occupational physician would be more correct than the answers given in a mailed questionnaire. For the interview, the physicians had a guide with the questions that should be asked, but it is assumable that they explained the question and asked for more details when necessary. Both the physicians who did the interviews were engaged in finding the "true" exposure of the participants. We thus assume that the interview information was more like an expert assessment which has been used as a gold standard in other studies [6,11].

In the period before this study was started, there had been some media attention on the possible health effects in dental nurses due to mercury exposure. Some recall bias with the possibilities of exaggeration of exposure among the dental nurses could be anticipated, while among the dentists, who had been the employers of the dental nurses, a recall bias in the other direction was possible. In this context we saw the possibility that the pairs of one dental nurse and one dentist would differ more in their judgement on the degree of mercury exposure than the pairs of two dental nurses. This was, however, not the case in this study. The greatest differences were in fact between pairs of two nurses at the same clinic. Thus, despite the media attention there does not seem to be any substantial differences in describing the methods used between dentists and dental nurses. There was, however, a marked difference between the pairs that had worked in the same clinic regarding the start and termination years for the different treatment methods, independent of the occupation. The mean difference between workers was, however, not different from the within worker mean difference on the one question that was possible to compare: the number of years with the use of a Dentomat. There was a marked difference in agreement between the dental nurses and the dentist in regard to the questions as to whether the amalgam was soft or firm. As it was the dental nurses in the clinics who filled the Dentomat and who prepared the amalgam for use before treatment, this was not very surprising. The dentists, however, were the ones who used the amalgam and were responsible for the conditions in the clinic. The agreement between two nurses in the same clinic was much better; 70% of the pairs of dental nurses agreed or partly agreed on these questions. In this between worker study, we chose for practical reasons to concentrate on responders from smaller cities and community centers. As we neither think that dental personnel from smaller cities or communities will have answered differently compared to dental personnel in bigger cities, nor that the preparation methods in use were systematically different between smaller and bigger communities we feel confident that the restrictions made did not lead to any decisive bias.

Regarding the agreement within workers, the question if the participants had ever used copper amalgam had a very good agreement based on the kappa statistics. In regard to the other methods, the agreement was moderate. The sensitivity of the questionnaire information, taking the answer at the interview to be true, was quite high for all preparation methods, while the specificity was somewhat lower. In our study, there were more false positive than false negative answers for all the three preparation methods when regarding the interview answers to be true. This is not quite in accordance with earlier studies that have reported the specificity to be higher than the sensitivity [11,12] and that mailed questionnaires tend to under-report exposure [4]. This is a particular concern in studies with a low prevalence of exposure where it is critical to avoid false positive exposure classification [2]. For instance, the specificity was 0.60 for our question that asked if the participants had ever mixed amalgam manually in a mortar. As the prevalence of this particular exposure was also low, this is a source of misclassification that could lead to serious bias. The specificity for the question about the use of the Dentomat was also low, but for this question, the prevalence of exposure was high and thus the concern less pronounced. We chose to interpret that the low specificity for ever having mixed amalgam manually in a mortar may be explained by some misunderstanding and unclear wording in the questionnaire. The method, "mixing amalgam manually in a mortar", was meant from our side to be the method used in the fifties and the sixties where metallic mercury and metal powder were weighed separately and merged together manually in the mortar. We are afraid that some of the respondents may have misinterpreted the question in the questionnaire and have regarded it as relating to the small mortar used to handle the amalgam subsequent to the mixing in a Dentomat or a capsule. In the interview, the question for this preparation method was much more specific. This shows the necessity that the wording of questions is clear and without possibilities for misunderstanding. This is particularly the case for exposures that are less common in the study group. Missing information on details regarding particular questions was a problem in this study because many single questions from the questionnaire were used together to calculate a relative exposure score. This was difficult because one or more single questions were not answered by most of the respondents. The results showed that the more detailed the questions were, the greater proportion of the respondents gave incomplete answers.

In this study where we had the opportunity to compare a limited number of answers from a questionnaire with interview data, we are quite aware of the limitation both in regard to the number of questions and the number of respondents. Still, we find the results worth reporting, and we also hope that they contribute a little to sought-after knowledge about the possibilities and limitations in retrospective exposure assessment using a questionnaire.

## Conclusions

The results of this study indicate that our mailed questionnaire caused misclassification of exposure. In this study the differences in describing the methods used was independent of occupational status but the prevalence of assumed false positive exposure classification was higher than false negative. This may result in serious bias if the prevalence of exposure is low. The necessity to be precise and correct when formulating the specific questions must be emphasized. Due to missing information detailed questionnaires may be inefficient if the goal is to construct exposure measures from combinations of many answers in the questionnaire.

## Competing interests

The authors declare that they have no competing interests.

## Authors' contribution

Both authors have participated in the design of the study and the drafting of the manuscript. KS has in addition performed the analyses. Both authors have read and approved the final manuscript.
